# Specific challenges posed by artificial intelligence in research ethics

**DOI:** 10.3389/frai.2023.1149082

**Published:** 2023-07-06

**Authors:** Sarah Bouhouita-Guermech, Patrick Gogognon, Jean-Christophe Bélisle-Pipon

**Affiliations:** ^1^School of Public Health, Université de Montréal, Montréal, QC, Canada; ^2^Centre de recherche, CHU Sainte-Justine, Montréal, QC, Canada; ^3^Faculty of Health Sciences, Simon Fraser University, Burnaby, BC, Canada

**Keywords:** artificial intelligence, AI ethics, normative guidance, research ethics, research ethics board

## Abstract

**Background:**

The twenty first century is often defined as the era of Artificial Intelligence (AI), which raises many questions regarding its impact on society. It is already significantly changing many practices in different fields. Research ethics (RE) is no exception. Many challenges, including responsibility, privacy, and transparency, are encountered. Research ethics boards (REB) have been established to ensure that ethical practices are adequately followed during research projects. This scoping review aims to bring out the challenges of AI in research ethics and to investigate if REBs are equipped to evaluate them.

**Methods:**

Three electronic databases were selected to collect peer-reviewed articles that fit the inclusion criteria (English or French, published between 2016 and 2021, containing AI, RE, and REB). Two instigators independently reviewed each piece by screening with Covidence and then coding with NVivo.

**Results:**

From having a total of 657 articles to review, we were left with a final sample of 28 relevant papers for our scoping review. The selected literature described AI in research ethics (i.e., views on current guidelines, key ethical concept and approaches, key issues of the current state of AI-specific RE guidelines) and REBs regarding AI (i.e., their roles, scope and approaches, key practices and processes, limitations and challenges, stakeholder perceptions). However, the literature often described REBs ethical assessment practices of projects in AI research as lacking knowledge and tools.

**Conclusion:**

Ethical reflections are taking a step forward while normative guidelines adaptation to AI's reality is still dawdling. This impacts REBs and most stakeholders involved with AI. Indeed, REBs are not equipped enough to adequately evaluate AI research ethics and require standard guidelines to help them do so.

## 1. Introduction

The twenty first century is often defined as the era of artificial intelligence (AI) Brynjolfsson and Andrew, 2017). For a long time, humans have been conceptualizing an autonomous entity capable of human-like functions and more. Many innovations have preceded what we now know as AI (Stark and Pylyshyn, 2020). The mathematical and computational progress has had a significant impact on what made today's AI possible and flourish so quickly in the span of the last few years (Calmet and John, 1997; Xu et al., 2021). Many place their bet on AI's potential to revolutionize most fields. As ubiquitous as it seems, AI's role in our society remains ambiguous. Although Artificial Intelligence comes in different forms, essentially, it is predisposed to simulate human intelligence (Mintz and Brodie, 2019). AI has many forms: voice or facial recognition applications or even medical diagnosis systems (radiology, dermatology, etc.), algorithms that increase user service, and more (Copeland, 2022). AI is mainly used to increase productivity and make tasks less burdensome. It has proven to absorb and analyze more data in a shorter period than humans. Indeed, some have noticed patients' satisfaction increasing, better financial performance, and better data management in healthcare (Davenport and Rajeev, 2018). Many innovations emanated from AI's ability to collect large sets of data which resulted in better predictions on different issues, helping to understand information collected throughout history, or depicting puzzling phenomena more efficiently (The Royal Society, The Alan Turing Institute, 2019).

However, advances made in AI come with concerns about ethical, legal, and social issues (Bélisle-Pipon et al., 2021). AI systems (AIS) are part of professionals' decision-making and occasionally take over that role, making us wonder how responsibilities and functions are divided between each participating party (Dignum, 2018). Another issue worth investigating is data bias. A group of individuals initially programs AI to adhere to a set of pre-established data. This data could already be biased (i.e., favoring one group of people over another based on their race or social-economic status) by having one specific group represented and marginalizing the rest (Müller, 2021). Another fundamental issue to consider is data privacy. People are worried about using their data, which has become easier to access by big companies (Mazurek and Karolina, 2019). It is now much more strenuous to track where all the existing information goes. The lack of transparency has decreased public's trust. Many, such as industry representatives, governments, academics, and civil society, are working toward building better frameworks and regulations to design, develop and implement AI efficiently (Cath, 2018). Considering the multidisciplinary aspect of AI, different experts are called to provide their knowledge and expertise on the matter (Bélisle-Pipon et al., 2022). Many fields must leave room to adjust their standard of practice. One field that will be discussed in this study is research ethics.

Research ethics boards (REBs; the term REB is used for simplicity and includes REC, Research ethics committees, and IRB, Institutional review boards) have been created to ensure that ethical practices are adequately followed during research projects to ensure participant protection and that advantages outweigh the induced harms (Bonnet and Bénédicte, 2009). To achieve this, they follow existent codes and regulations. For instance, REBs in Canada turn to the *Canadian Tri-Council Policy Statement (TCPS2)* to build their framework in research ethics. In contrast, the US uses the *US Common Rule* as a model (Page and Jeffrey, 2017). Many countries have a set of guidelines and laws that are used as a starting point to set boundaries for AI use. However, ordinances and regulations regarding AI are limited (O'Sullivan et al., 2019). The lack of tools makes it harder for REBs to adjust to the new challenges created by AI. This gap reflects the need to understand better the current state of knowledge and findings in research ethics regarding AI.

To inform and assist REBs in their challenges with AI, we conducted a scoping review of the literature on REBs' current practices and the challenges AI may pose during their evaluation. Specifically, this article aims to raise the issues and good practices to support REBs' mission in research involving AI. To our knowledge, this is the first review on this topic. After gathering and analyzing the relevant articles, we will discuss the critical elements in research ethics AI while considering REBs' role.

## 2. Methodology

**Figure 1:**
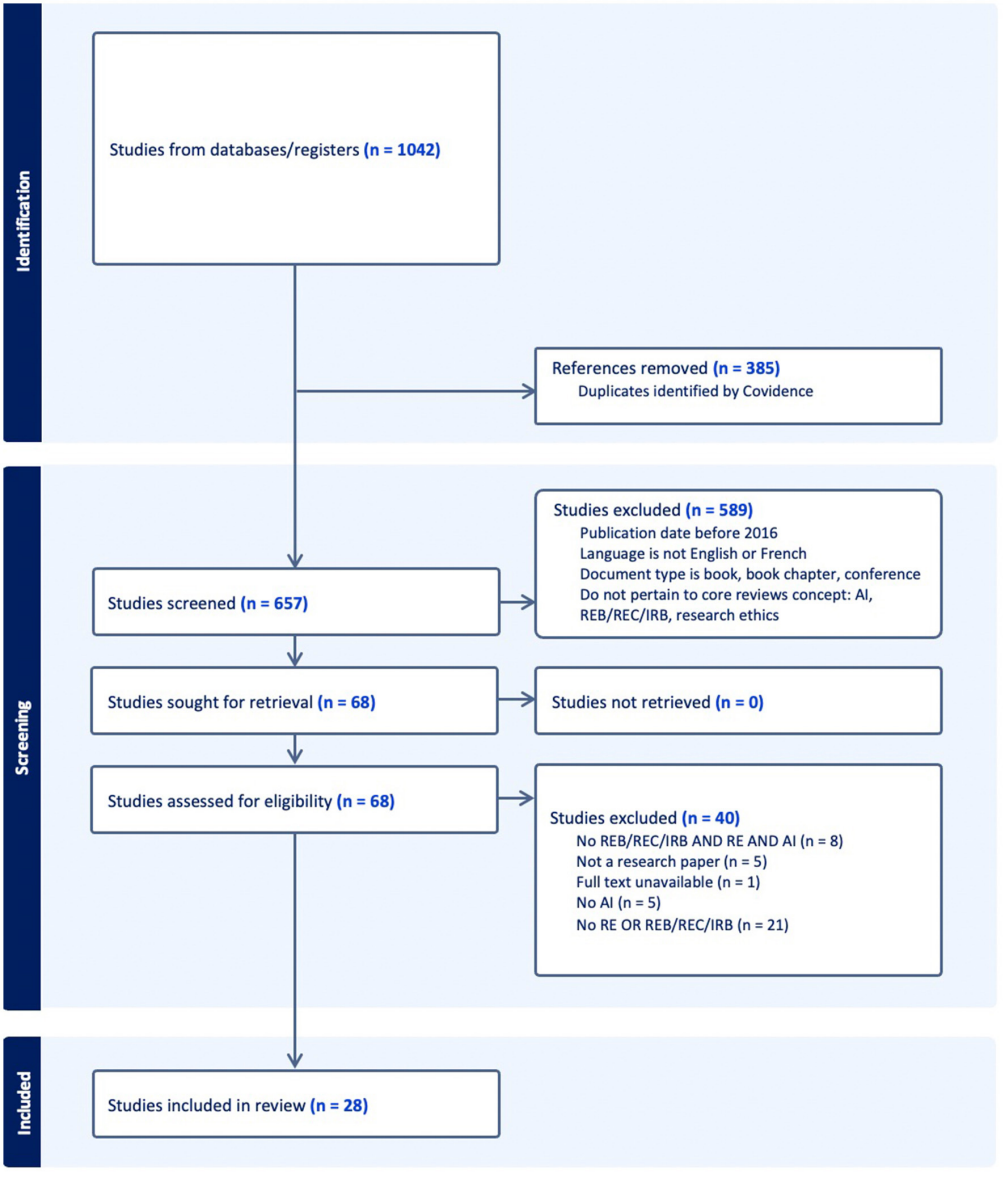
PRISMA Flowchart. AI, Artificial intelligence; REB, Review ethics board; REC, Research ethics committees; IRB, Institutional review boards; RE, Research ethics.

To better understand the REBs' current practices toward AI in research, we conducted a scoping review on articles generated from PubMed, Ovid, and Web of Science. Since the literature behind our research question is still preliminary, opting for a scoping review seemed like the better approach to garner the existing and important papers related to our topic (Colquhoun et al., 2014). A scoping review was preferred over a systematic review since the studied field is not yet clearly defined, and the literature behind it is still very limited (Munn et al., 2018). After a preliminary overview of relevant articles which showcased the limited literature on the matter, we opted for a scoping review for a more exploratory approach. A scoping review will allow us to collect and assess essential information from the emerging literature and gather it into one place to help advance future studies. We focused on two concepts: AI and REB. Table 1 of this article presents equations for each concept that differ from one search engine to another. We sought to use general terms frequently used in the literature to define both concepts. After validating the research strategy with a librarian, the subsequent articles were imported to Covidence. The criteria exclusion to determine whether studies were not eligible for the review were: articles published before 2016, articles published in a language other than English or French, studies found in books, book chapters, or conferences, and studies that did not contain AI, REB, and research ethics. The criteria inclusion to determine whether studies were eligible for the review were (as seen in Table 2): articles published between 2016 and 2021, articles published in English or French, studies published in a peer-reviewed article, a commentary, an editorial, a review or a discussion paper and studies containing AI, REB and research ethics. We have chosen 2016 as the starting year of the review because while it was a year that showed significant advancement in AI, many were concerned about its ethical implications (Mills, 2016; Stone et al., 2016; Greene et al., 2019). Since AI is fast evolving, the literature from the recent years was used to obtain the emergent and most recent results (Nittas et al., 2023; Sukums et al., 2023). Figure 1 presents our review flowchart following PRISMA's guidelines (Moher et al., 2009). The initial total number of studies subject to review was 657. For the first step of the review, two investigators screened all 657 articles by carefully reviewing their titles and abstracts and considering the inclusion and exclusion criteria. That resulted in excluding 589 irrelevant studies leaving us with 68 studies. In the next review step, two investigators did a full-text reading of the studies assessed for eligibility. This Full-Text review excluded 40 studies (21 articles with no “research ethics” or “research ethics committee,” eight papers with no “REB,” “RE,” and “AI,” five articles with no “Artificial Intelligence,” five pieces that were not research papers and one unavailable full text). With NVivo (Braun and Victoria, 2006), each article was analyzed according to a set of different themes that aimed to answer the questions of the current topic. “REB” is used throughout the article as an umbrella term to include all the variations that are used to label research ethics boards in different countries.

**Table 1:** Search strategy.

**Concepts**	**Terms**
**AI**	**PB** **=** (“artificial intelligence” OR “AI” OR “ambient intelligence” OR “Machine Learning” OR “Deep Learning” OR “machine intelligence” OR “Natural Language Processing^*^” OR bot OR robot^*^ or “computational intelligence” or “computer reasoning” or “computer vision system^*^”)) OR (“Artificial Intelligence” or “Machine Learning” [MeSH Terms]) **EMB** **=** exp Artificial Intelligence/ or (artificial intelligence or “AI” or ambient intelligence or Machine Learning or Deep Learning or machine intelligence or Natural Language Processing^*^ or bot or robot^*^ or computational intelligence or computer reasoning or computer vision system^*^).ab,kf,kw,ti. **WoS** **=** (“artificial intelligence” or “AI” or “ambient intelligence” or “Machine Learning” or “Deep Learning” or “machine intelligence” or “Natural Language Processing^*^” or bot or robot^*^ or “computational intelligence” or “computer reasoning” or “computer vision system^*^”)
**REB**	**PB** **=** (“research ethic^*^” or “responsible research” or “REB” or “IRBS” or “Institutional Review Board^*^” or “Ethical review board^*^” or “ERB” or ((“Ethics committee^*^” or “Ethic committee^*^”) adj2 (research^*^ or independent)) OR (“Ethics” or “Research Ethics Committee” or Research^*^[MeSH Terms]) **EMB** **=** ethics committees/ or ethics committees, research/ or ethics, research/ or (research ethic^*^ or responsible research or “REB” or “IRBS” or Institutional Review Board^*^ or Ethical review board^*^ or “ERB” or ((Ethics committee^*^ or Ethic committee^*^) adj2 (research^*^ or independent))).ab,kf,kw,ti. **WoS** **=** (“research ethic^*^” or “responsible research” or “REB” or “IRBS” or “Institutional Review Board^*^” or “Ethical review board^*^” or “ERB” or ((“Ethics committee^*^” or “Ethic committee^*^”) NEAR/2 (research^*^ or independent)))

PB, PubMed; EMB, Embase; WoS, Web of Science.

**Table 2:** Selection criteria.

	**Description**
Date	2016–2021 (5 years)
Language	English; French
Type of publication	Peer-reviewed article, a commentary, an editorial, a review, or a discussion paper
Concepts	AI, REB, and research ethics

## 3. Results

**Figure 2:**
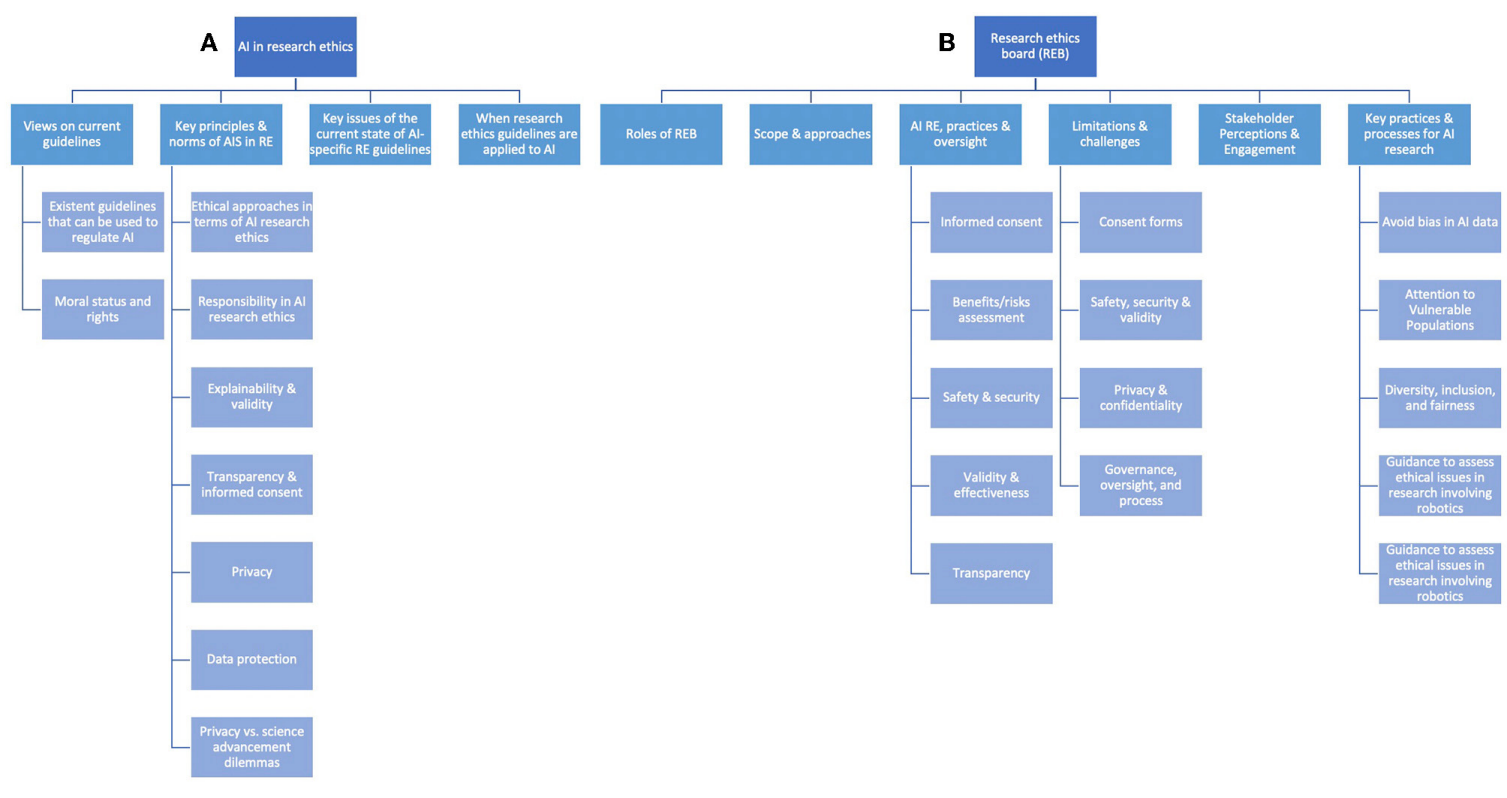
Architecture that illustrates the article's results structure starting with the two main domains: **(A)** AI and research ethics and **(B)** research ethics boards.

The following section includes the results based on the thematic coding grid used to create the different sections relevant to our topic (see Figure 2). The results come from our final sample of articles.

### 3.1. AI and research ethics

Researchers are faced with several ethical quandaries while navigating research projects. They are urged to safeguard human research participants' protection when working with human research participants. However, it is not always simple to balance the common good (i.e., develop solutions for the wider population) and the individual interest (i.e., research participants' safety) (Ford et al., 2020; Battistuzzi et al., 2021). Researchers are responsible for anticipating and preventing risks from harming participants while advancing scientific knowledge, which requires maintaining an adequate risk-benefit ratio (Sedenberg et al., 2016; Ford et al., 2020). With AI's fast growth, another set of issues is added to the existing ones: data governance, consent, responsibility, justice, transparency, privacy, safety, reliability, and more (Samuel and Derrick, 2020; Gooding and Kariotis, 2021). This section will describe the views on current guidelines to regulate AI, key principles and ethical approaches, and the main issues. In the current climate, we expect continuity on the following concepts: responsibility, explainability, validity, transparency, informed consent, justice, privacy, data governance, benefits and risks assessment, safety, and justice.

#### 3.1.1. Views on current guidelines

##### 3.1.1.1. Existent guidelines that can be used to regulate AI

The current normative guidelines do not make up for the few AI-related guidelines (Aymerich-Franch and Fosch-Villaronga, 2020). However, in addition to the ethical standards used as a basis for AI use guidelines, the UN published a first set of guidelines to regulate AI (Chassang et al., 2021). Many projects, like the Human Brain Project (HBP), took the initiative to encourage discussions from different parties to anticipate issues that could result from their research (Stahl and Coeckelbergh, 2016; Aicardi et al., 2018, 2020). Researchers and developers can access tools that help orient their reflections on their responsible use of technology (Aymerich-Franch and Fosch-Villaronga, 2020). Furthermore, the implementation of ethical approval committees (i.e., Human Research Ethics Committees in Australia) that uses a soft-governance model, which leans toward ethical regulation and is less restrictive than legal regulations, would help prevent studies or companies abuse their participants or users (Andreotta et al., 2021). Many are contemplating using digital health ELSI to encourage the implementation of ethical standards in AI when laws and regulations are lacking in it (Nebeker et al., 2019).

Articles have mentioned many leading countries in AI research. Supplementary Table 1 showcases the progress and effort the European Union (EU) and other countries have made regarding AI regulation. The countries, alongside the EU, that were often mentioned throughout our final sample were the following: Australia, Canada, China, the European Union, the United Kingdom, and the United States. Since this information is strictly from our selected articles, some information was unavailable. While noticeable progress is being made regarding AI development and regulation, most countries have shown little indication, if any, of AI research ethics.

##### 3.1.1.2. Moral status and rights

While guidelines and norms are shifting to fit AI standards, many questions on moral status and rights are raised to adapt to this new reality. Authors argue that we cannot assign moral agency to AI and robots. There are multiple reasons for it: robots do not seem capable of solving problems ethically (Stahl and Coeckelbergh, 2016), AI's lack of explanation regarding its generated results, and the absence of willingness to choose (Farisco et al., 2020) which might impact decision making in research ethics.

Rights are attributed to different living entities. For instance, in the EU, the law protects animals as sentient living organisms and unique tangible goods. Their legal status also obliges researchers not to harm animals during research projects, making us question the status and rights we should assign AIS or robots (Chassang et al., 2021). Indeed, Miller pointed out that having a machine at one's disposal raises questions on human-machine relationships and the hierarchical power it might induce (Miller, 2020).

#### 3.1.2. Key principles and norms of AI systems in research ethics

We have seen that the lexicon and the language used invoke both classical theories and contextualization of AI ethics benchmarks within the practices and ethos of research ethics.

##### 3.1.2.1. Ethical approaches in terms of AI research ethics

The literature invoked the following classic theories: the Kantian-inspired model, utilitarianism, principlism (autonomy, beneficence, justice, and non-maleficence), and the precautionary principle. Table 3 illustrates these essential ethical approaches found in our final sample, along with their description in terms of AI research ethics.

**Table 3:** Critical key ethical approaches that were raised in the present scoping review and their description in terms of AI research ethics.

**Key ethical approaches**	**Description in terms of AI research ethics**
**Kantian-inspired model**	The Kantian approach demands that researchers act responsibly during research (Jacobson et al., 2020). The same procedure should be executed to ensure responsible AI. *Ex: AI developers must ensure that their system is adequate and will not cause harm for society. Researchers must responsibly use AI systems during their projects*.
**Utilitarianism**	The utilitarianism approach focuses on consequences and the best outcome for most people. It is invoked in the dilemma of using machine learning algorithms to help progress science and maintain participants' privacy (Jacobson et al., 2020). *Ex: AI systems should serve the wellbeing of participants and other individuals over their usage for scientific progress*.
**Principlism**	Principlism is an approach that underlines principles such as autonomy, beneficence, non-maleficence, and justice invoked in issues raised while developing and using machine learning (Jacobson et al., 2020).
Autonomy	Participants' autonomy suggests they can consent to their own will when participating in a research project using AI (Grote, 2021). *Ex: Many concerns are raised about the eventuality that AI becomes fully autonomous, which takes away our control over them* (Aicardi et al., 2020). *Some may even say they should be granted moral autonomy* (Farisco et al., 2020). *Although, for now, AI mainly relies on humans, whether the users, employers, or programs which then brings up the notion of responsibility* (Chassang et al., 2021). *While it may not be autonomous, its purpose is to assist humans, which could negatively impact our autonomy* (McCradden et al., 2020c).
Beneficence	AI is more efficient in specific tasks than humans, bringing better results for those involved (Grote, 2021). *Ex: One of AI's benefits is that it can generate more precise and accurate results* (Ienca and Ignatiadis, 2020). *AI can also search data more efficiently and make predictions* (Andreotta et al., 2021; Grote, 2021). *Furthermore, robots can assist humans in alleviating them from specific tasks* (Battistuzzi et al., 2021).
Justice	AI's use should be done in a way that does not put people at a disadvantage (Nebeker et al., 2019). *Ex: Data bias can result from the under-representation of minority groups which may lead to algorithmic discrimination disadvantaging the groups in question in receiving the proper treatment of care* (Ienca and Ignatiadis, 2020; Jacobson et al., 2020; Grote, 2021; Li et al., 2021).
Non-maleficence	*AI must distinguish right from wrong to ensure non-maleficence* (Farisco et al., 2020). *Ex: Robots should not cause harm* (Stahl and Coeckelbergh, 2016).
**Precautionary principle**	The precautionary principle in AI may serve as a guiding framework to encourage responsible AI research and development, prioritizing the protection of individuals, society, and the environment from potential negative impacts that may arise from AI systems (Chassang et al., 2021). *Ex: AI developers should consider societal needs and ensure that potential risks will be taken care of at the beginning of product conception. Governments should put in place regulations to prevent future harm with AI from happening*.

##### 3.1.2.2. Responsibility in AI research ethics

Public education and ethical training implementation could help governments spread awareness and sensitize people regarding research ethics in AI (Cath et al., 2018). Accountability of AI regulation and decision-making should not strictly fall into stakeholders' hands but also be based on solid legal grounds (Chassang et al., 2021). Digital mental health apps and other institutions will now be attributed responsibilities that have usually been acclaimed to professionals or researchers using the technology (i.e., decision-making, providing users with enough tools to understand and use products, being able to help when needed, etc.) (Gooding and Kariotis, 2021). Scientists and AI developers must not throw caution to the wind regarding the possibility that biased algorithms could be fed to AI models (Ienca and Ignatiadis, 2020). Clinicians will have to tactfully manage to inform patients of the results generated by machine learning (ML) models while considering their risk of error and bias (Jacobson et al., 2020). It is still vague to attribute responsibility to specific actors. However, it is necessary to have different groups work together to tackle the problem (Meszaros and Ho, 2021; Samuel and Gemma, 2021). Some consider that validity, explainability, and open-source AI systems are some of the defining points that lead to responsibility. With the advancement of technologies and its gain of interest, the sense of social responsibility also increased. Indeed, every actor must contribute to making sure that these novel technologies are developed and used in an ethical matter (Nebeker et al., 2019; Aicardi et al., 2020).

##### 3.1.2.3. Explainability and validity

An important issue with AIS usually raised is the explainability of results. Deep learning (DL) is another type of ML with more extensive algorithms that encloses data with a broader array of interpretations (Chassang et al., 2021). This makes it harder to explain how DL and AI models reached a particular conclusion (Ienca and Ignatiadis, 2020; Jacobson et al., 2020). This poses transparency issues that are challenging to participants (Grote, 2021).

Since AI is known for its ‘black-box' aspect, where results are difficult to justify, it is difficult to fully validate a model with certainty (Ienca and Ignatiadis, 2020). Deciding to monitor research participants closely could help validate results which, in theory, would bring more accurate results. However, close monitoring could also have a negative effect by influencing participants' decisions based on whether they mind being monitored or not. This event could, as a result, produce more inaccurate results (Jacobson et al., 2020). Furthermore, it could be more challenging in certain contexts to promote validity when journals and funding bodies favor new and innovative studies over ethical research on AI, even if the latter is being promoted (Samuel and Gemma, 2021).

##### 3.1.2.4. Transparency and informed consent

According to the White House Office of Science and Technology Policy (OSTP), transparency would help solve many ethical issues (Cath et al., 2018). Transparency allows research participants to be aware of a study's different outlooks and comprehend them (Sedenberg et al., 2016; Grote, 2021). The same goes for new device users (Chassang et al., 2021). AI models (i.e., products, services, apps, sensor-equipped wearable systems, etc.) produce a great deal of data that does not always come from consenting users (Ienca and Ignatiadis, 2020; Meszaros and Ho, 2021). Furthermore, AI's black-box imposes a challenge to obtain informed consent since the lack of explainability of AI-generated results might not allow participants to have enough information to give out their informed consent (Jacobson et al., 2020; Andreotta et al., 2021). Thus, it is essential to make consent forms easy to understand for the targeted audience (Nebeker et al., 2019).

However, the requirement to get informed consent could lead to other less desirable implications. Some argue that requiring authorization for all data, especially studies that hold a vast set of data, might lead to data bias and a decrease in data quality because it only entices a specific group of people to give out consent which leaves out a significant part of the population (Ford et al., 2020).

##### 3.1.2.5. Privacy

While the levels of privacy differ from one scholar to another, the concept of privacy remains a fundamental value to human beings (Andreotta et al., 2021). Through AI and robotics, data can be seen as attractive commodities which could compromise privacy (Cath et al., 2018). Researchers are responsible for keeping participants unidentifiable while using their data (Ford et al., 2020). However, data collected from many sources can induce a higher risk of identifying people. While pursuing their research study, ML researchers still struggle to comply with privacy guidelines.

###### 3.1.2.5.1. Data protection

According to a study, most people do not think data protection is an issue. One reason to explain this phenomenon is that people might not fully grasp the magnitude of its impact (Coeckelbergh et al., 2016). Indeed, the effect could be very harmful to some people. For instance, data found about a person could decrease their chances of employment or even of getting insurance (Jacobson et al., 2020). Instead of focusing on data minimization, data protection should be prioritized to ensure ML models get the most relevant data, ensuring data quality while maintaining privacy (McCradden et al., 2020b). Another point worth mentioning is that the GDPR allows the reuse of personal data for research purposes, which might allow companies who wish to pursue commercial research to bypass certain ethical requirements (Meszaros and Ho, 2021).

###### 3.1.2.5.2. Privacy vs. science advancement dilemmas

Some technology-based studies face a dichotomy between safeguarding participants' data and making scientific advancements. This does not always come easily since ensuring privacy can compromise data quality, while studies with more accurate data usually lead to riskier privacy settings (Gooding and Kariotis, 2021). Indeed, with new data collection methods in public and digital environments, consent and transparency might be overlooked for better research results (Jacobson et al., 2020).

#### 3.1.3. Key issues of the current state of AI-specific RE guidelines

Many difficulties have arisen with the soaring evolution of AI. There has been a gap between research ethics and AI research, inconsistent standards regarding AI regulation and guidelines, and a lack of knowledge and training in these new technologies has been widely noticed. Medical researchers are more familiar with research ethics than computer science researchers and technologists (Nebeker et al., 2019; Ford et al., 2020). This shows a disparity in knowledge between different fields.

With new technologies comes the difficulty in assessing them (Aicardi et al., 2018; Aymerich-Franch and Fosch-Villaronga, 2020; Chassang et al., 2021). Research helps follow AI's progress and ensures it does so responsibly and ethically (Cath et al., 2018). Unfortunately, applied and research ethics are not always in sync (Gooding and Kariotis, 2021). AI standards mostly rely on ethical values rather than concrete normative and legal regulations, which have become insufficient (Samuel and Derrick, 2020; Meszaros and Ho, 2021). The societal aspects of AI are more discussed amongst researchers than the ethics part of research (Samuel and Derrick, 2020; Samuel and Gemma, 2021).

Many countries have taken the initiative to regulate AI using ethical standards. However, guidelines vary from one region to another. It has become a strenuous task to establish a consensus of strategies, turn principles into laws, and make them practical (Chassang et al., 2021). It does not only come down to countries that have differing points of views but journals as well. Indeed, validation for an AI research project publication could differ from one journal to another (Samuel and Gemma, 2021). Even though ethical, legal, and social implications (ELSI) are used to help oversee AI, regulations and AI-specific guidelines remain scarce (Nebeker et al., 2019).

#### 3.1.4. When research ethics guidelines are applied to AI

While there is a usual emphasis that is being made on ethical approbation for research projects, there are other projects that are not required to follow an ethics guideline. In the United Kingdom, some research projects do not require ethics approval (i.e., social media data, geolocation data, anonymous secondary health data with an agreement) (Samuel and Derrick, 2020). A study highlighted that most papers gathered that used available data from social media did not have an ethical approbation (Ford et al., 2020). Some technology-based research projects ask for consent from their participants but skip requesting ethical approval from a committee (Gooding and Kariotis, 2021). Some non-clinical research projects are exempt from an ethics evaluation (Samuel and Gemma, 2021). Tools do not always undergo robust testing before validation either (Nebeker et al., 2019). Of course, ethical evaluation remains essential in multiple other settings: when minors or people lacking capacity to make an informed decision are involved, when users are recognizable, when researchers seek users' data directly (Ford et al., 2020), when clinical data or applications are used (Samuel and Gemma, 2021), etc.

### 3.2. Research ethics board

Historically REBs have focused on protecting human participants in research (e.g., therapeutic, nursing, psychological, or social research) from complying with the requirements of funding or federal agencies like NIH or FDA (Durand, 2005). This approach has continued, and in many countries, REBs are fundamentally essential to ensure that research involving human participants is conducted in compliance with ethics guidelines and national and international regulations.

#### 3.2.1. Roles of REB

The primary goal of REBs focuses on reviewing and overseeing research to provide the necessary protection for research participants. REBs consist of groups of experts and stakeholders (clinicians, scientists, community members) who review research protocols with an eye toward ethical concerns. They ensure that protocols comply with regulatory guidelines and can withhold approval until such matters have been addressed. Also, they were designed to play an anticipatory role, predicting what risks might arise within research and ironing out ethical issues before they appeared (Friesen et al., 2021). Accordingly, REBs aim to assess whether the proposed research project meets specific ethical standards regarding the foreseeable impacts on human subjects. However, REBs are less concerned with the broader consequences of research and its downstream applications. Instead, they focus on the direct effects on human subjects during or after the research process (Prunkl et al., 2021). Within their established jurisdiction, REBs can develop a review process independently. Considering the specific context of AI research, REBs would aim to mitigate the risks of potential harm possibly caused by technology. This could be done by reviewing scientific questions relating to the origin and quality of the data, algorithms, and artificial intelligence; confirming the validation steps conducted to ensure the prediction models work; requesting further validation to be carried out if required (Samuel and Derrick, 2020).

#### 3.2.2. Scope and approaches

AI technologies are rapidly changing health research; these mutations might lead to significant gaps in REB oversight. Some authors who analyzed these challenges suggest an adaptative scope and approach. To achieve an AI-appropriate research ethics review, it is necessary to clearly define the thresholds and characteristics of cardinal research ethics considerations, including what constitutes a “*human participant*, what is a *treatment*, what is a *benefit*, what is a *risk*, what is considered a *publicly available information*, what is considered an *intervention in the public domain*, what is a *medical data*, but also what is *AI research”* (Friesen et al., 2021).

There is an urgent need to tailor the technology and its development, evaluation, and use contexts (i.e., digital mental health) (Gooding and Kariotis, 2021). Health research involving AI features requires intersectoral and interdisciplinary participatory efforts to develop dynamic, adaptive, and relevant normative guidance. It also requires practice navigating the ethical, legal, and social complexities of patient data collection, sharing, analysis, interpretation, and transfer for decision-making in a natural context (Gooding and Kariotis, 2021). Also, these studies imply multi-stakeholder participation (such as regulatory actors, education, and social media).

This diversity of actors seems to be a key aspect in this case. Still, it requires transparent, inclusive, and transferable normative guidance and norms to ensure that all understand each other and meet the normative demands regarding research ethics. Furthermore, bringing together diverse stakeholders and experts is worthwhile, especially when the impact of research can be significant, difficult to foresee, and unlikely to be understood by any single expert, as with AI-driven medical research (Friesen et al., 2021). In this stake, several factors are beneficial to promote cooperation between academic research and industry: inter-organizational trust, collaboration experience, and the breadth of interaction channels. Partnership strategies like collaborative research, knowledge transfer, and research support may be essential to embolden this in much broader terms than strict technology transfer (Aicardi et al., 2020).

#### 3.2.3. AI research ethics, practices, and governance oversight

According to the results of our review, REBs must assess the following seven considerations of importance during AI research ethics review: (1) Informed consent, (2) benefit-risks assessment, (3) safety and security, (4) validity and effectiveness, (5) user-centric approach and design, (6) transparency. In the literature, some authors have pointed out specific questions about considerations REBs should be aware of. The following Table 4 reports the main highlights REBs might rely on.

**Table 4:** Main highlights for the reviewed body of literature (divided by key salient ethical considerations).

**Concepts**	**Identified issues**	**Key reviewed articles on this issue**	**Key insights and best practices for supporting research ethics stakeholders**
Scope and approaches	Intersectoral and interdisciplinary participatory efforts are needed to develop dynamic, adaptative, and relevant normative guidance and practices	Gooding and Kariotis, 2021	Work up new ways of collaboration for REBs.
Diversity and fair representation	Concerns regarding inclusion are often found in RCTs.	Grote, 2021	Relatively little data is found for other types of research. Does not seem to consider research projects with retrospective data.
Biases toward vulnerable population	AI systems are either fed with actual biased data or generate biased results.	Cath et al., 2018; Chassang et al., 2021; Grote, 2021	If the algorithms are not considered unbiased and representative of the general population, results could exclude minorities and, thus, be harmful.
Informed consent	Transparency and accessibility of relevant information could help participants better understand a situation which will allow them to make a conscious decision. For informed consent, there should be a focus on the impacts and risks that arise from interventions using AI and ML. There is a dilemma between giving out all the information to participants and only the relevant ones they need.	Sedenberg et al., 2016; Nebeker et al., 2019; Grote, 2021 Jacobson et al., 2020 Jacobson et al., 2020	The issue of informed consent raises concerns about the following: - Nature of the information may be disclosed in the consent while the model is still at the preliminary stage of development - What risks can be revealed when we do not know the impacts of the technology? Possibility of causing harm to participants if incomplete or unreliable information is disclosed. The problem of the quality of consent and its scope in a complex and rapidly evolving technological field. Questioning the need to develop new tools for consent. Limits on the possibility of revoking consent compared to other types of research. REBs must make sure that researchers are giving intelligible information to participants.
Benefit risks assessment	*One trigger point is to establish whether involvement of AI in RCT improves the standard of care. Many factors need to be considered to justify conducting AI RCTs, to risks research participants imposed in studies. Fine subject selection and equal distribution of risks and benefits across different populations must also be considered. Determine risk threshold. Data monitoring and management of the risks intervention requirements (full assessment, based only on study data, etc.) Management of passively collected data (e.g., the content of text messages) vs. predictive algorithms is still under development*.	Grote, 2021 Jacobson et al., 2020	REBs should clearly define how AI may reduce the trial burden and improve the benefit-risk ratio in a research project. REBs should ensure that participants are not at a higher risk of being part of a minority in a population. REBs should identify and recommend appropriate measures to mitigate the specific risks that are embedded in AI in RCT.
Safety and security (End user-centered)	The research project and technologies used should not pose any harm to participants. These issues should be evaluated according to users' perspectives; the assessment should consider the reality's context. REB's lack of understanding of AI models makes assessing their impacts on safety difficult. Measures should be established to counteract negative impacts. Anticipate the implications of AI use (human protection, legal act, etc.)	Coeckelbergh et al., 2016 Coeckelbergh et al., 2016 Nebeker et al., 2019 Chassang et al., 2021	Adequate risk mitigation for a new technology implies that the REB has a good knowledge of the technology and its impacts REBs and researchers should identify adverse effects of AI systems and their consequences that may harm participants; identify mechanisms to repair potential harms The possibility that the harm is physical or moral can be an issue
Transparency	This concept will become a challenge with AI's black box, making it difficult to explain each result generated.	Jacobson et al., 2020; Andreotta et al., 2021	Ensure that the research project is explained in a way that is understandable to participants.
Privacy and confidentiality	Confusion between governance and confidentiality protection mechanisms. Greater emphasis on governance to the detriment of specific considerations for confidentiality or other ethical issues. Scientific advancement and data quality could impact individuals' privacy by collecting data extensively and transferring them.	Samuel and Gemma, 2021 Samuel and Gemma, 2021 Jacobson et al., 2020; Gooding and Kariotis, 2021	Pay more attention to algorithm and software development, allowing to broaden analysis and ethical evaluation toward ethical considerations toward privacy and confidentiality. Questioning the limits of current anonymization techniques with the use of AI systems. Questioning the new harms that may result from breaches of privacy and violations of confidentiality. Develop mechanisms to prevent, limit, and, if necessary, repair the damage resulting from these new potential breaches of privacy and confidentiality.
Justice, equity, and fairness	Standard of fair representation	Grote, 2021	Results do not mention the distribution of research benefits from these technologies. To address this issue, REBs should: -Focus more on these issues to rebalance their approach, which is more centered on governance. -Put AI systems and their potential into question to reduce inequalities and strengthen health equity Access to research benefits should be investigated to ensure a return of individual results. Challenges of transmitting general results to the community.
Validity and effectiveness	Consensus to appreciate the normative implications of AI technologies: -technology development -application of technology in real-time conditions The challenging aspect of understanding black box	McCradden et al., 2020c Ienca and Ignatiadis, 2020	REBs do not currently have an effective method to evaluate the validity of results generated by AI. REBs need the right tools to ensure that the expected aims of AI systems are achievable. The development of the system should meet the concrete needs of the populations targeted by the technology In an actual situation, the potential for transformation of the practice and the care offered must be ensured. Adaptations can be complex; in practice, modifications to the protocol are more difficult considering the nature of AI.

##### 3.2.3.1. Informed consent

Some authors argue that the priority might be to consider whether predictions from a specific machine learning model are appropriate for informing decisions about a particular intervention (Jacobson et al., 2020). Others advocate carefully constructing the planned interventions so research participants can understand them (Grote, 2021).

The extent to which researchers should provide extensive information to participants is not evident among stakeholders. So far, research suggests that there is no clear consensus among patients on whether they would want to know this kind of information about themselves (Jacobson et al., 2020). Hence the question remains whether patients want to see if they are at risk, mainly if they cannot be told why, as factors included in machine learning models generally cannot be interpreted as having a causal impact on outcomes (Jacobson et al., 2020). Therefore, sharing information from an uninterpretable model may adversely affect a patient's perception of their illness, confuse them, and immediate concerns about transparency.

##### 3.2.3.2. Benefits/risks assessment

The analysis of harms and potential benefits is critical when assessing human research. REBs are well concerned with this assessment to prevent unnecessary risks and promote benefits. Considerations of the potential benefits and harms to patient-participants are necessary for future clinical research, and REBs are optimally positioned to perform this assessment (McCradden et al., 2020c). Additional considerations like benefit/risk ratio or effectiveness and the systematic process described previously are necessary. Risk assessments could have a considerable impact in research involving mobile devices or robotics because preventive action and safety measures may be required in the case of imminent risks. Thus, REB risk assessment seems very important (Jacobson et al., 2020).

Approaching AI research ethics through user-centered design can represent an interesting avenue to understand better how REB can conduct risk/benefices assessment. For researchers, involving users in the design of AI research is likely to promote better research outcomes. Hence, this can be reached by investigating how AI research is actually meeting users' needs and how this may generate intended and unintended impacts on them (Chassang et al., 2021; Gooding and Kariotis, 2021). Indeed there is insufficient reason to believe that AI research will produce positive benefits unless it is evaluated with a focus on patients and situated in the context of clinical decision-making (McCradden et al., 2020c). Consequently, REBs might focus on the broader societal impact of this research (Chassang et al., 2021).

##### 3.2.3.3. Safety and security

Safety and security are significant concerns for AI and robotics, and their assessment may rely on end-users' perspectives. To address the safety issue, it is not sufficient for robotics researchers to say that their robot is safe based on literature and experimental tests. It is crucial to find out about the perception and opinions of end-users of robots and other stakeholders (Coeckelbergh et al., 2016). Testing technology in real-life scenarios is vital for identifying and adequately assessing technology's risks, anticipating unforeseen problems, and clarifying effective monitoring mechanisms (Cath et al., 2018). On the other hand, there is a potential risk that an AIS misleads the user in realizing a legal act.

##### 3.2.3.4. Validity and effectiveness

Validity is a crucial consideration and one on which there is consensus to appreciate the normative implications of AI technologies. To this end, it is necessary for research ethics that researchers' protocols be explicit about many elements and describe their validation model and performance metrics in a way that allows for assessment of the clinical applicability of their developing technology (McCradden et al., 2020b). In addition, in terms of validity, simulative models have yet to be appropriately compared with standard medical research models (including *in vitro, in vivo*, and clinical models) to ensure they are correctly validated and effective (Ienca and Ignatiadis, 2020). Considering many red flags raised in recent years, AI systems may not work equally well with all sub-populations (racial, ethnic, etc.). Therefore, AI systems must be validated for different subpopulations of patients (McCradden et al., 2020b).

Demonstration of value is essential to ensure the scientific validity of the claims made for technology but also to attest to the proven effectiveness once deployed in a real-world setting and the social utility of a technology (Nebeker et al., 2019). When conducting a trial for the given AI system, the main interest should be to assess its overall reliability, while the interaction with the clinician might be less critical (Grote, 2021).

##### 3.2.3.5. Transparency

Transparency entails understanding how technology behaves and establishing thresholds for permissible (and impermissible) usages of AI-driven health research. Transparency requires clarifying the reasons and rationales for the technology's design, operation, and impacts (Friesen et al., 2021). Identified risks should be accompanied by detailed measures intended to avoid, reduce, or eliminate the risks. The efficiency of such efforts should be assessed upstream and downstream as part of the quality management process. As far as possible, testing methods, data, and assessment results should be public. Transparent communication is essential to make research participants, as well as future users aware of the technology's logic and functioning (Chassang et al., 2021).

The implications presented in Table 4. Seem to encourage REBs to adopt a more collaborative approach to grasp a better sense of reality in different fields. The analysis also showed that data bias is a flagrant problem whether AI is used or not and that this discriminatory component should be taken care of to avoid emphasizing the problem with AI. Informed consent is another value that REBs prioritize and will have to be adapted to AI because new information might have to be disclosed to participants. Safety and security are always essential to consider. However, other measures will be implemented with AI to ensure that participants are not set in danger. One of the main aspects of AI is data sharing and the risk that this might breach participants' privacy. The methods put in place now might not be suitable for AI's fast evolution. The questions of justice, equality, and fairness that have not been resolved in our current society will also have to be instigated in the AI era. Finally, the importance of validity was raised numerous times. Unfortunately, REBs do not have the right tools to evaluate AI. It will be necessary for AI to meet the population's needs. Furthermore, definitions of specific values and principles that REBs usually respond to will have to be reviewed and adapted according to AI.

#### 3.2.4. Limitations and challenges

Our results point to several discrepancies between the critical considerations for AI research ethics and REB review of health research and AI/ML data.

##### 3.2.4.1. Consent forms

According to our review, there is a disproportionate focus on consent before other ethical issues. Authors argue that the big piece the REBs ask for relies on consent, not the AI aspect of the project. This finding suggests that narrowing AI research ethics around consent concerns remains problematic. In some stances, the disproportionate focus on consent, along with the importance REBs place on consent forms and participant information sheets, has settled how research ethics is defined, e.g., viewed as a proxy for ethics best practice, or in some cases, as an ethics panacea (Samuel and Gemma, 2021).

##### 3.2.4.2. Safety, security, and validity

Authors report a lack of knowledge for safety review. It appears clear that REBs may not have the experience or expertise to conduct a risk assessment to evaluate the probability or magnitude of potential harm. Similarly, the training data used to inform the algorithm development are often not considered to qualify as human subjects research, which – even in a regulated environment – makes a prospective review for safety potentially unavailable (Nebeker et al., 2019).

On the other hand, REBs lack appropriate assessing processes for assessing whether AI systems are valid, effective, and apposite. The requirement to evaluate the evidence of effectiveness adds to a range of other considerations with which REBs must deal (i.e., the protection of participants and the fairness in the distribution of benefits and burdens). Therefore, there is still much to be done to equip REBs to evaluate the effectiveness of AI technologies, interventions, and research (Friesen et al., 2021).

##### 3.2.4.3. Privacy and confidentiality

Researchers point to a disproportionate focus on data privacy and governance before other ethical issues in medical health research with AI tools. Focus on privacy and data governance issues warrants further attention, as privacy issues may overshadow other issues. Indeed it seems problematic and led to a narrowing of ethics and responsibility debates being perpetuated throughout the ethics ecosystem, often at the expense of other ethical issues, such as questions around justice and fairness (Samuel and Gemma, 2021). REBs appear to be less concerned about the results themselves. One explained that when reviewing their AI-associated research ethics applications, REBs focus more on questions of data privacy than other ethical issues, such as those related to the research and the research finding. Others painted a similar picture of how data governance issues were a centralized focus when discussing their interactions with their REB. According to these stakeholders, REBs focus less on the actual algorithm than how the data is handled, and the issue remains around data access and not about the software (Samuel and Gemma, 2021).

##### 3.2.4.4. Governance, oversight, and process

Lack of expertise appears to be a significant concern in our results. Indeed, even when there is oversight from a research ethics committee, authors observe that REB members often lack the experience or confidence regarding particular issues associated with digital research (Samuel and Derrick, 2020).

Some authors advocate that ML researchers should complement the membership of REBs since they are better situated to evaluate the specific risks and potential unintended harms linked to the methodology of ML. On the other hand, REBs should be empowered to fulfill their role in protecting the interests of patients and participants and enable the ethical translation of healthcare ML (McCradden et al., 2020c). However, we can notice that researchers expressed different views about REBs' expertise. While most acknowledged a lack of AI-specific proficiency, for many, this remains straightforward because the ethical issues of their AI research were nonexceptional compared to other ethics issues raised by “big data” (Samuel and Gemma, 2021).

Limits of process and regulation are another concern faced by REBs, including a lack of consistency in decision-making within and across REBs, a lack of transparency, poor representation of the participants and public they are meant to represent, insufficient training, and a lack of measures to examine their effectiveness (Friesen et al., 2021). There are several opinions on the need for and the effectiveness of REBs, with critics lamenting excessive bureaucracy, lack of reliability, inefficiency, and, importantly, high variance in outcomes (Prunkl et al., 2021). To address the existing gap of knowledge between different fields, training could be used to help rebalance this and ensure sufficient expertise for all research experts to pursue responsible innovation (Stahl and Coeckelbergh, 2016).

Researchers described the lack of standards and regulations for governing AI at the level of societal impact; the way that ethics committees in institutions work is still acceptable. But there is a need for another level of thinking that combines everything and does not look at one project simultaneously (Samuel and Gemma, 2021).

Finally, researchers have acknowledged the lack of ethical guidance, and some REBs report feeling ill-equipped to keep pace with rapidly changing technologies used in research (Ford et al., 2020).

#### 3.2.5. Stakeholder perceptions and engagement

Researchers' perspectives on AI research ethics may vary. While some claim that researchers often take action to counteract the adverse outcomes created by their research projects (Stahl and Coeckelbergh, 2016), others promulgate that researchers do not always notice these outcomes (Aymerich-Franch and Fosch-Villaronga, 2020). When the latter occurs, researchers are pressed to find solutions to deal with those outcomes (Jacobson et al., 2020).

Furthermore, researchers are expected to engage more in AI research ethics. Researchers must demonstrate cooperation with certain institutions (i.e., industries and governments) (Cath et al., 2018). Researchers are responsible for ensuring that their research project is conducted responsibly by considering participants' needs (Jacobson et al., 2020). Usually, research ethics consist of different researchers coming from multidisciplinary fields who are better equipped to answer further ethical and societal questions (Aicardi et al., 2018). However, there could be a clash of interests between parties while setting goals for a research project (Battistuzzi et al., 2021).

A lot of the time, different stakeholders do not necessarily understand other groups' realities. Therefore, research is vital to ensure that stakeholders can understand one another and be in the same scheme of things. This will help advance AI research ethics (Nebeker et al., 2019).

**Figure 3:**
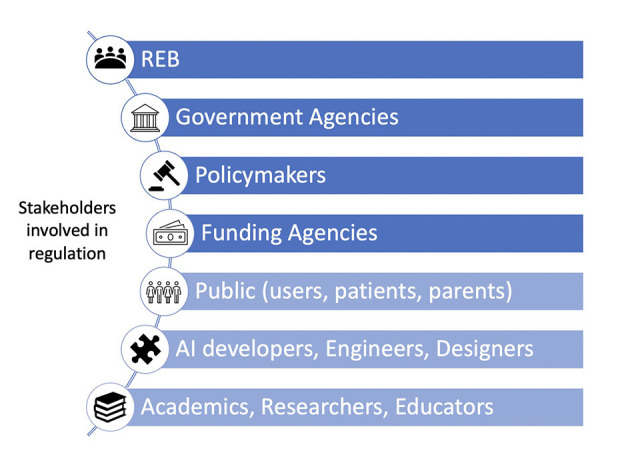
Overview of the stakeholders involved in regulation regarding AI in research ethics: the main active stakeholders (dark blue) and the main passive stakeholders (light blue).

Responsibility for ensuring a responsible utilization of AI lies within various groups of stakeholders (Chassang et al., 2021). Figure 3 portrays some of these groups often mentioned throughout the literature. This figure aims to illustrate the amount and variety of stakeholders needed to collaborate to ensure using AI in a responsible matter.

Many others, such as the private sector, can be added to the list. Studies have shown that private companies' main interest is profit over improving health with the data collected using AI (McCradden et al., 2020a). Another problematic element with the private sector: they do not often fall under the regulation of ethical oversight boards, which means that AI systems or robots that come from private companies do not necessarily follow an accepted ethical guideline (Sedenberg et al., 2016). This goes beyond ethical research concerns.

#### 3.2.6. Key practices and processes for AI research

REBs may face new challenges in the context of research involving AI tools. Authors are calling for specific oversight mechanisms, especially for medical research projects.

##### 3.2.6.1. Avoid bias in AI data

While AI tools provide new opportunities to enhance medical health research, there is an emerging consensus among stakeholders regarding bias concerns in AI data, particularly in clinical trials. Since bias can worsen pre-existing disparities, researchers should proactively target a wide range of participants to establish sufficient evidence of an AI system's clinical benefit across different populations. To mitigate selection bias, REBs may require randomization in AI clinical trials. To achieve this, researchers must start by collecting more and better data from social minority groups (Grote, 2021). Also, awareness of biases concerns should be taken into account in the validation phase, where the performance of the AI system gets measured in a benchmark data set. Hence it is crucial to test AI systems for different subpopulations. Therefore, affirmative action in recruiting research participants in AI RCTs deems us ethically permissible (Grote, 2021). However, authors reported that stakeholders might encounter challenges accessing needed data in a context where severe legal constraints are imposed on sharing medical data (Grote, 2021).

##### 3.2.6.2. Attention to vulnerable populations

Vulnerable populations require excellent protection against risks they may face in research.

When involving vulnerable populations, such as those with a mental health diagnosis, in AI medical health research, additional precautions should be considered to ensure that those involved in the study are duly protected from harm – including stigma and economic and legal implications. In addition, it is essential to consider whether access barriers might exclude some people (Nebeker et al., 2019).

##### 3.2.6.3. Diversity, inclusion, and fairness

Another issue, which needs to be raised when considering critical practices and scope in AI research, relates to fair representation, diversity, and inclusion. According to Grote, one should explore concerns for the distribution of research participants and representatives for the state, country, or even world region in which the AI system gets tested. Here the author advocates if we should instead aim for a parity distribution of different gender, racial and ethnic groups. Hence, he raised several questions to support the reflection of REB on diversity, inclusion, and fairness issues: How should the reference classes for the different subpopulations be determined? Also, what conditions must be met for fair subject selection in AI RCTs? And finally, when, if ever, is it morally justifiable to randomize research participants in medical AI trials? (Grote, 2021).

##### 3.2.6.4. Guidance to assess ethical issues in research involving robotics

The aging population and scarcity of health resources are significant challenges healthcare systems face today. Consequently, people with disabilities, especially elders with cognitive and mental impairments, are the most affected. The evolving field of research with assistive robots may be useful in providing care and assistance to these people. However, robotics research requires specific guidance when participants have physical or cognitive impairments. Indeed particular challenges are related to informed consent, confidentiality, and participant rights (Battistuzzi et al., 2021). According to some authors, REBs should ask several questions to address these issues: Is the research project expected to enhance the quality of care for the research participants? What is/are the ethical issue/s illustrated in this study? What are the facts? Is any important information not available in the research? Who are the stakeholders? Which course of action best fits with the recommendations and requirements set out in the “Ethical Considerations” section of the study protocol? How can that course of action be implemented in practice? Could the ethical issue/s presented in the case be prevented? If so, how? (Battistuzzi et al., 2021).

Which ethical and social issues may neurorobotics raise, and are mechanisms currently implemented sufficiently to identify and address these? Is the notion that we may analyze, understand and reproduce what makes us human rooted in something other than reason (Aicardi et al., 2020)?

##### 3.2.6.5. Understanding of the process behind AI/ML data

A good understanding of the process behind AI/ML tools might be of interest to REBs when assessing the risk/benefit ratio of medical research involving AI. However, there seems to be a lack of awareness of how AI researchers gain results. Authors argue that it would not be impossible to induce perception about the external environment in the neuron culture and to interpret the signals from the neuron culture as motor commands without a basic understanding of this neural code (Bentzen, 2017). Indeed, when using digital health technologies, the first step is to ask whether the tools, be they apps or sensors, or AI applied to large data sets, have demonstrated value for outcomes. One should ask whether they are clinically effective, or if they measure what they purport to measure (validity) consistently (reliability), and finally, if these innovations also improve access to those at the highest risk of health disparities (Nebeker et al., 2019).

Indeed, the ethical issues of AI research raise major questions within the literature. What may seem surprising at first sight is that the body of literature is still relatively small and appears to be in an embryonic state regarding the ethics of the development and use of AI (outside the scope of academic research). The literature is thus more concerned with the broad questions of what constitutes research ethics in AI-specific research and with pointing out the gaps in normative guidelines, procedures, and infrastructures adapted to the oversight of responsible and ethical research in AI. Perhaps unsurprisingly, most of the questions related to study within the health sector. This is to be expected given the ascendancy of health in general within the research ethics field (Faden et al., 2013). Thus, most considerations relate to applied health research, the implications for human participants (whether in digital health issues, research protocols, or interactions with different forms of robots), and whether projects should be subject to ethics review.

Specifically in AI-specific research ethics, interestingly, traditional issues of participant protection (including confidentiality, consent, and autonomy in general) and research involving digital technologies intersect and are furthered by the uses of AI. Indeed, as AI requires big data and behaves very distinctly from other technologies, the primary considerations raised by the body of literature studied were predominantly classical in AI ethics but contextualized and exacerbated within research ethics practices. For instance, one of the most prevalent ethical considerations raised and discussed was privacy and the new challenges regarding the massive amount of data collected and its use. If a breach of confidentiality were to happen or data collection would lead to discovering further information, this would raise the possibility of harming individuals (Ford et al., 2020; Jacobson et al., 2020). In addition, informed consent was widely mentioned and focused on transparency and explainability when the issues were AI-specific. Indeed, AI's black-box issue of explainability was raised many times. This is a challenge because it is not always easy to justify the results generated by AI (Jacobson et al., 2020; Andreotta et al., 2021). This then poses a problem with transparency. Indeed, participants expect to have the necessary information relevant to the trial to make an informed and conscious decision regarding their participation. Not having adequate knowledge to share with participants might not align with informed consent.

Furthermore, another principle was brought up many times, which was responsibility. Responsibility is shared chiefly between the researcher and the participant (Gooding and Kariotis, 2021). Now that AI is added to the equation, it has become harder to determine who strictly should be held accountable for the occurrence of certain events (i.e., data error) and in what context (Meszaros and Ho, 2021; Samuel and Gemma, 2021). While shared responsibility is an idea many share and wish to implement, it is not easy. Indeed, as seen in Figure 3, many stakeholders (e.g., lawmakers, AI developers, AI users) may participate in responsibility sharing. However, much work will have to be put into finding a fair way to share responsibility between each party involved.

## 4. Discussion

**Figure 4:**
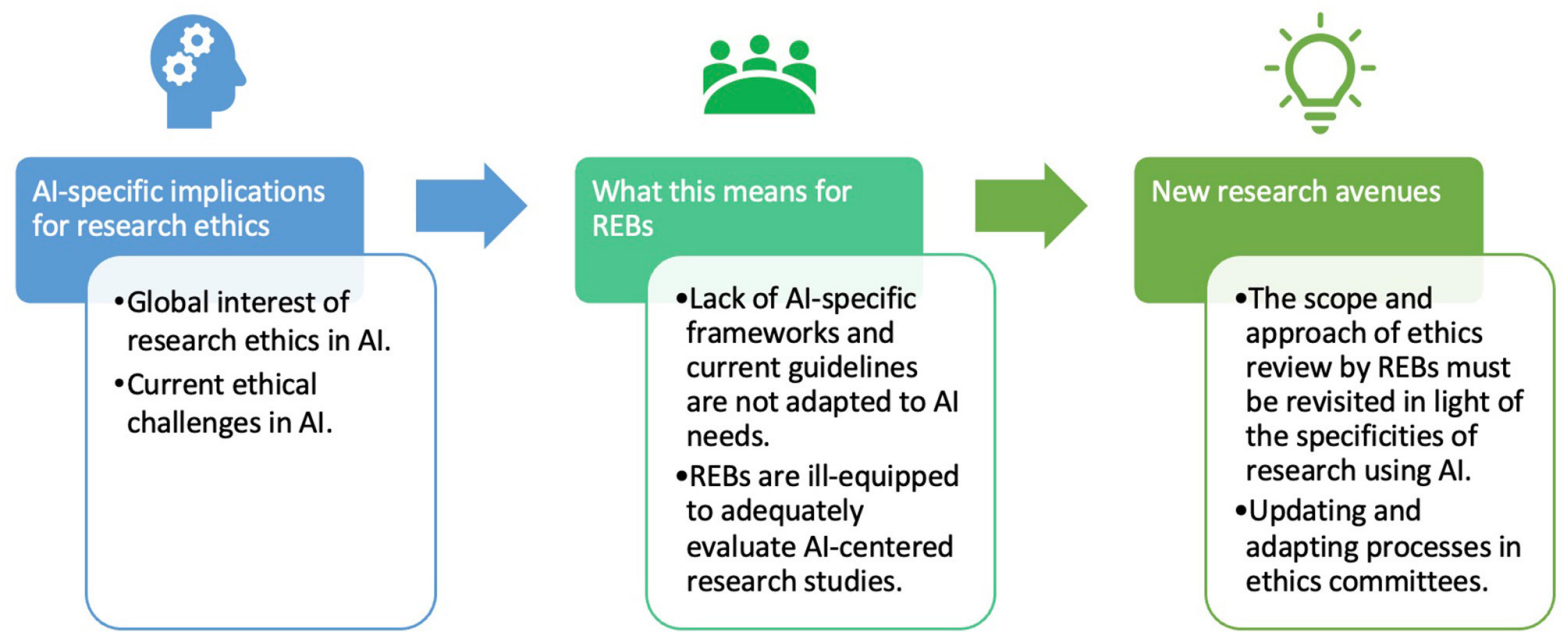
Line of progression on AI ethics resolution in research.

Our results have implications mainly on three levels as shown in Figure 4. Indeed, AI-specific implications for research ethics is first addressed followed by REBs who take on these challenges. Finally, new research avenues are discussed before ending with the limitations.

### 4.1. AI-specific implications for research ethics

The issues raised by AI are eminently global. It is interesting to see in the articles presented in the scoping review that researchers in different countries are asking questions colored by the jurisdictional, social, and normative context in which the authors work. However, there appears to be heterogeneity in the advancement of AI research ethics thinking; this is particularly evident in the progress of research ethics initiatives within countries (see Supplementary Table 1). A striking finding is that very little development has been done regarding AI-specific standards and guidelines to frame and support research ethics worldwide.

At this point, the literature does not discuss the content of norms and their application to AI research. Instead, it makes initial observations about AI's issues and challenges to research ethics. In this sense, it is possible to see that the authors indicate new challenges posed by the emergence of AI in research ethics. AI makes many principles more challenging to assess (it seems quite difficult to use the current guidelines to balance the risks and benefits). One example is that it has become unclear which level of transparency is adequate (Geis et al., 2019). AI validity, on the other hand, is not always done in an optimal manner throughout AI's lifecycle (Vollmer et al., 2020). Accountability remains a continuing issue since it is still unclear who to hold accountable and to what extent with AI in play (Greatbatch et al., 2019). In addition, AI is also known to amplify certain traditional issues in research ethics. For example, AI blurs the notion of free and informed consent since the information a patient or participant needs regarding AI is yet to be determined (Gerke and Timo Minssen, 2020). Privacy's getting harder to manage because it has become possible with AI to identify individuals by analyzing all the data available, even after deidentification (Ahuja, 2019). Data bias is another leading example where AI would not necessarily detect data bias it's being fed but could also generate more biased results (Auger et al., 2020).

Interestingly, the very distinction between the new AI-related issues and the old, amplified ones is still not entirely clear to researchers. For instance, while AI is quickly targeted for generating biased results, the source of the problem could come from biased data fed to AI (Cath et al., 2018; Chassang et al., 2021; Grote, 2021). Another issue is the lack of robustness, where it is challenging to rely entirely on AI to always give accurate results (Grote, 2021). However, this issue is also found in human-based decision-making. Thus, the most efficient use of AI could depend on context. The final decision could be reserved for humans limiting AI's role as an assistive tool (Ienca and Ignatiadis, 2020). Therefore, drawing a picture of what is new and less so is difficult. However, there is no doubt that AI is disrupting the field of research ethics, its processes, practices, and standards. This also points to the fact that no AI-specific Research Ethics Guidelines can help give a sense of how best to evaluate AI in a compatible way with RE guidance.

Another observation is that research ethics (and a fortiori research ethics committees) are very limited in their approach to AI development and research. This means that research ethics only comes into play at a specific point in developing AI technologies, interventions, and knowledge, i.e., after developing an AIS and before its implementation in a real context. Thus, research ethics, understood as it has been developed in most countries, focuses on what happens within public organizations and when human participants are involved. This excludes technological developments developed by industry and does not require ethical certification. Therefore, the vast majority of AIS outside the health and social services sector will not be subject to research ethics reviews, such as data found in social media or geolocation (Samuel and Derrick, 2020). But even within the health sector, AIS that do not directly interact with patients could largely be excluded from the scope of research ethics and the mandate of REBs. This makes the field of AI research ethics very new and small compared to responsible AI innovation.

### 4.2. What this means for REBs

No author seems to indicate that REBs are prepared and equipped to evaluate research projects involving AI as rigorously, confidently, and consistently as for more traditional research protocols (i.e., not involving AI). One paper from Sedenberg et al. (2016) expressively indicates that the current REB model should be replicated in the private sector to help oversee and guide AI development (Sedenberg et al., 2016). Arguably the call is mostly about adding an appraising actor to private sector technology developments than praising REBs for their mastery and competence in AI research ethics review. Yet, it still holds a relatively positive perception of the current readiness and relevance of REBs to research ethics. This may also reflect a lack of awareness (from uninformed stakeholders) of the limitations faced by REBs, which on paper can probably be seen as being able to evaluate research protocols involving AI and other projects. This is, however, disputed or refuted by the rest of the literature studied.

The bulk of the body of literature reviewed was more circumspect about the capacity of REBs. Not that they are not competent, but rather that they do not have the tools to start with a normative framework relevant to AI research, conceptually rigorous and comprehensive, and performative and appropriate to the mandates, processes, and practices of REBs. Over the last several decades, REBs have primarily relied on somewhat comprehensive and, to some extent, harmonized, regulations and sets of frameworks to inform and guide their ethical evaluation. The lack therefore, REBs face new challenges without any tools to support them with their decisions on AI dilemmas. The authors of our body of literature thus seem to indicate a higher expectation on all stakeholders to find solutions to address the specificities and challenges of AI in research ethics.

One of the first points is quite simple: determining when research involving AI should be subject to research ethics review. This simple point and observation is not consensual. Then, we can raise some serious concerns about the current mandate of REBs and the ability to evaluate AI with their current means and framework. Not only are they missing clear guidelines to do any kind of standard assessments on AI in research ethics, but they are also missing clearly defined roles on their account. Indeed, should their role be extended to look not just at research but also at the use of downstream technology? Or does this require another ethics oversight body that would look more at the technology in real life? This raises the question of how a lifecycle evaluative process can best be structured and how a continuum of evaluation can be developed that is adapted to this adaptive technology.

### 4.3. New research avenues

After looking at the heterogeneity of norms and regulations regarding AI in different countries, there should be an interest in initiating an international comparative analysis. The aim would be to investigate how REBs have adapted their practices in evaluating AI-based research projects without much input and support from norms. This analysis could raise many questions (i.e., could there be key issues that are impossible to universalize?).

#### 4.3.1. The scope and approach of ethics review by REBs must be revisited in light of the specificities of research using AI

The primary considerations we discuss above raise new challenges on the scope and approaches of REB practices when reviewing research with AI. Furthermore, applications developed within the research framework often rely on a population-based system, leading REBs to question whether their assessment should focus on a systematic individual approach rather than societal considerations and their underlying foundations.

However, AI research is still emerging, underlining the difficulties of completing such a debate. Finally, one can wonder about the importance of current guidelines in AI within the process of ethical evaluation by the REBs. Should this reflection be limited only to the REBs? Or should it include other actors meaning scientists or civil society?

AI ethics is not limited to research. While it is less discussed, AI ethics raises many existential questions. Dynamics such as the patient-physician relationship will have to adapt to a new reality (Chassang et al., 2021). With human tasks being delegated to AI, notions of personhood (Aymerich-Franch and Fosch-Villaronga, 2020), autonomy (Aicardi et al., 2020), and human status in our society (Farisco et al., 2020) are threatened. This leads to delving into the question of “what it is to be human?”. Robots used in therapies aimed to care for patients (i.e., autistic children) could induce attachment issues and other psychological impacts (Coeckelbergh et al., 2016). This projects another issue: AI overreliance, a similar problem brought up by current technological tools (i.e., cell phones) (Holte and Richard, 2021).

#### 4.3.2. Updating and adapting processes in ethics committees

AI ethics is still an emerging field. The REBs ensure the application of ethical frameworks, laws, and regulations. Our results suggest that research in AI involves complex issues that emerge around these new research strategies and methodologies using technologies such as computer science, mathematics, or digital technology. Thus, REBs' concerns remain on recognizing and assessing ethical issues that arise from these studies and adapting to rapid changes in this emerging field.

In research ethics, respect for a person's dignity is essential. In several normative frameworks, i.e., the TCPS in Canada, it means respect for persons, concern for wellbeing, and justice. In AI research, REBs might need to reassess the notion of consent or the participant's place in the study. As with all research, REBs must ensure informed consent. However, there does not seem to be a clear consensus on the standard for providing informed consent in AI research. For example, REBs should consider the issue of AI's interpretability in a research consent form; to translate transparent and intelligible results.

Another issue that REBs consider here is the role of participants in AI research. Indeed, active participant involvement is not always necessary in AI research to complete the data collection to meet the research objectives. It is often the case when data collection is completed from connected digital devices or by querying databases. However, the consequences amplified the phenomenon of dematerialization of research participation while facilitating data circulation.

Furthermore, AI research and the use of these new technologies call on REBs to be aware of the changes this implies for the research participant, particularly concerns such as the continuous consent process, management of withdrawal, or the duration of participation in the research.

While protecting the individual participant takes center stage in the evaluation of REBs, research with AI may focus more on using data obtained from databases held by governments, private bodies, institutions, or academics. In this context, should concerns for societal wellbeing prevail over the wellbeing of the individual? There does not appear to be a clear consensus on what principles should be invoked to address this concern.

### 4.4. Limitations

The focal point of AI evaluation was often about privacy protection and data governance, not AI's ethics. While data protection and governance are massively essential issues, it should be equally important to investigate AI issues, not to leave out concerns that should be dealt with, such as AI validity, explainability, and transparency. In addition, FAIR and the ethics of care, which are starting to become standard approaches in the field, were not invoked in the articles to inform AI ethics in research. This might be due to the study's lack of literature on AI ethics compared to research ethics in general.

Another limitation worth outlining is that our final sample mainly reflected the reality and issues found in healthcare, despite having a scoping review open to all fields using AI. This could be due to the fact that AI is becoming more prominent in the field of healthcare (Davenport and Kalakota, 2019). The field is also often linked to the development and presence of research ethics boards (Edwards and Tracey Stone, 2007). Having healthcare outshine the rest of the fields in our sample could also be attributed to research ethics mostly stemming from multiple medical research incidents throughout history (Aita and Marie-Claire, 2005).

Furthermore, throughout the studied articles, few to none mentioned countries were non-affluent. This poses concerns about widening disparities between developed and developing countries. Therefore, it is vital to acknowledge the asymmetry of legislative and societal norms between countries to better serve their needs and avoid colonized practices.

Finally, this topic lacks maturity. This study primarily shows that REBs cannot find guidance from the literature. Indeed, there is a scarcity of findings in the literature regarding recommendations and practices to adopt in research using AI. There are even fewer findings that specifically aim to equip REBs. Reported suggestions are often about privileged behavior that governments or researchers should adopt rather than establishing the proper criteria REBs should follow during their assessments. Therefore, this study does not lead to findings directly applicable to REBs practice and should not be used as a tool for REBs.

## 5. Conclusion

Every field has its ethical challenges and needs. The results in this article have shown this reality. Indeed, we've navigated through some of AI ethics general issues before investigating AI ethics research-specific issues. This led us to discern what research ethics boards focus on more adeptly during their evaluations and the limits imposed on them when evaluating AI ethics in research. While AI is a promising field to explore and invest in, many caveats force us to develop a better understanding of these systems. With AI's development, many societal challenges will come our way, whether they are current ongoing issues, new AI-specific ones, or those that remain unknown to us. Ethical reflections are taking a step forward while normative guidelines adaptation to AI's reality is still dawdling. This impacts REBs and most stakeholders involved with AI. However, throughout the literature, many suggestions and recommendations were provided. This could allow us to build a framework with a clear set of practices that could be implemented for real-world use.

## Author contributions

SBG: data collection, data curation, writing—original draft, and writing—review and editing. PG: conceptualization, methodology, data collection, writing—original draft, writing—review and editing, supervision, and project administration. JCBP: conceptualization, methodology, data collection, writing—review and editing, supervision, project administration, and funding acquisition. All authors contributed to the article and approved the submitted version.
