# Supplementary material for: Specific challenges posed by artificial intelligence in research ethics
Source: Front Artif Intell. 2023 Jul 6;6:1149082. doi: 10.3389/frai.2023.1149082 (PMC10358356; doi:10.3389/frai.2023.1149082)
Supplement: Supplementary file 1 [file Table_1.docx]

Supplementary Material

Challenges research ethics boards face during the evaluation of research projects using artificial intelligence while illustrating their current practices and guidelines – Scoping Review

First Author*, Co-Author, Co-Author

*** Correspondence:** Corresponding Author: email@uni.edu

# Supplementary Figures and Tables

TableS1. Guidelines, norms, and laws cited by the review literature on AI governance in different countries

| **Countries/international organizations** | **Highlights** | |
| --- | --- | --- |
|  | **AI governance** | **AI research ethics** |
| **Australia** | - The Australian Competition and Consumer Commission (ACCC) concluded that Australia’s *Privacy Act 1988* is not robust enough to ensure data protection (Andreotta, Kirkham, and Rizzi 2021). | NA |
| **Canada** | - Alongside France, Canada launched the Global Partnership on Artificial Intelligence (GPAI) to continue discussing AI regulations (Chassang et al. 2021). | NA |
| **China** | NA | - Research in China has fewer restrictions than in the EU and the US and is influenced by its government (Meszaros and Ho 2021). |
| **European Union (EU)** | - The EU established the European General Data Protection Regulation (GDPR) to protect data and privacy (Andreotta, Kirkham, and Rizzi 2021; Battistuzzi et al. 2021). - The European Parliament’s Committee on Legal Affairs (JURI) report suggests reassessing the EU’s current regulations and readjusting them to AI and robotics needs without being defined by expectations set outside of the EU (Cath et al. 2018). - The EU put in place another initiative to regulate AI, the Artificial Intelligence High-Level Expert Group (AIHLEG) guidelines that stakeholders could use (Chassang et al. 2021). | NA |
| **United Kingdom (UK)** | - The UK put in place many groups to help establish frameworks for AI use (i.e., the National Institute for Health and Care Excellence, the Medicines and Healthcare products Regulatory Agency, and the Centre for Data Ethics and Innovation) (Samuel and Derrick 2020). | NA |
| **United States (US)** | - The US has a Health Insurance Portability and Accountability Act (HIPAA) which helps protect health data (Battistuzzi et al. 2021). - The US also has its Food and Drug Administration (FDA) adjusting to AI algorithm usage (Jacobson et al. 2020). | - AI research is mainly used by the private sector in the US (Meszaros and Ho 2021). Without being limited to them, self-regulating approaches, like the Benefit People and Society AI partnership, incite the private sector to investigate AI ethics (Cath et al. 2018). |
